# Targeted Transvenous Embolization Under Sinus Balloon Protection for Transverse-Sigmoid Sinus Dural Arteriovenous Fistula: A Technical Note

**DOI:** 10.7759/cureus.93450

**Published:** 2025-09-28

**Authors:** Kenji Fukuda, Kodai Matsuda, Yuki Kozaki, Shota Sakai, Hiroshi Abe

**Affiliations:** 1 Department of Neurosurgery, Hakujyuji Hospital, Fukuoka, JPN; 2 Department of Neurology, Hakujyuji Hospital, Fukuoka, JPN; 3 Neurological Surgery, Fukuoka University Hospital, Fukuoka, JPN

**Keywords:** dural arteriovenous fistula, onyx, sinus balloon protection, transvenous embolization, transverse-sigmoid sinus

## Abstract

Dural arteriovenous fistulas (DAVFs) at the transverse-sigmoid sinus (TSS) are often treated with transarterial embolization (TAE) or sinus occlusion. Although sinus balloon protection has become increasingly utilized in TAE to preserve sinus patency, its application in transvenous embolization (TVE) has rarely been reported. In a case involving a Borden type Ⅰ/Cognard IIa TSS DAVF with localized parasinus shunting, three-dimensional rotational angiography clearly delineated the detailed structure of the shunted pouch, allowing precise transvenous access. TVE was successfully performed using coils and Onyx under sinus balloon protection, resulting in complete occlusion of DAVF while maintaining sinus patency. Targeted TVE under sinus balloon protection may offer a viable, sinus-preserving alternative to conventional TAE or sinus occlusion in select cases of TSS DAVFs with parasinus shunt concentrations.

## Introduction

Transverse-sigmoid sinus (TSS) dural arteriovenous fistulas (DAVFs) have complex vascular anomalies and can cause various symptoms ranging from benign tinnitus to life-threatening cerebral hemorrhage [1,2]. Endovascular treatment has become the first treatment for managing these lesions. Recent advancements in endovascular techniques have introduced sinus balloon protection as a method of maintaining venous sinus patency during transarterial embolization (TAE), particularly in DAVFs classified as Borden types Ⅰ and Ⅱ [3,4]. However, the use of sinus balloon protection in transvenous embolization (TVE) is extremely rare and limited. We report a case of TSS DAVF in which targeted TVE at the shunted pouch was performed under sinus balloon protection, and complete shunt occlusion was achieved with sinus patency.

## Technical report

**Figure 1:**
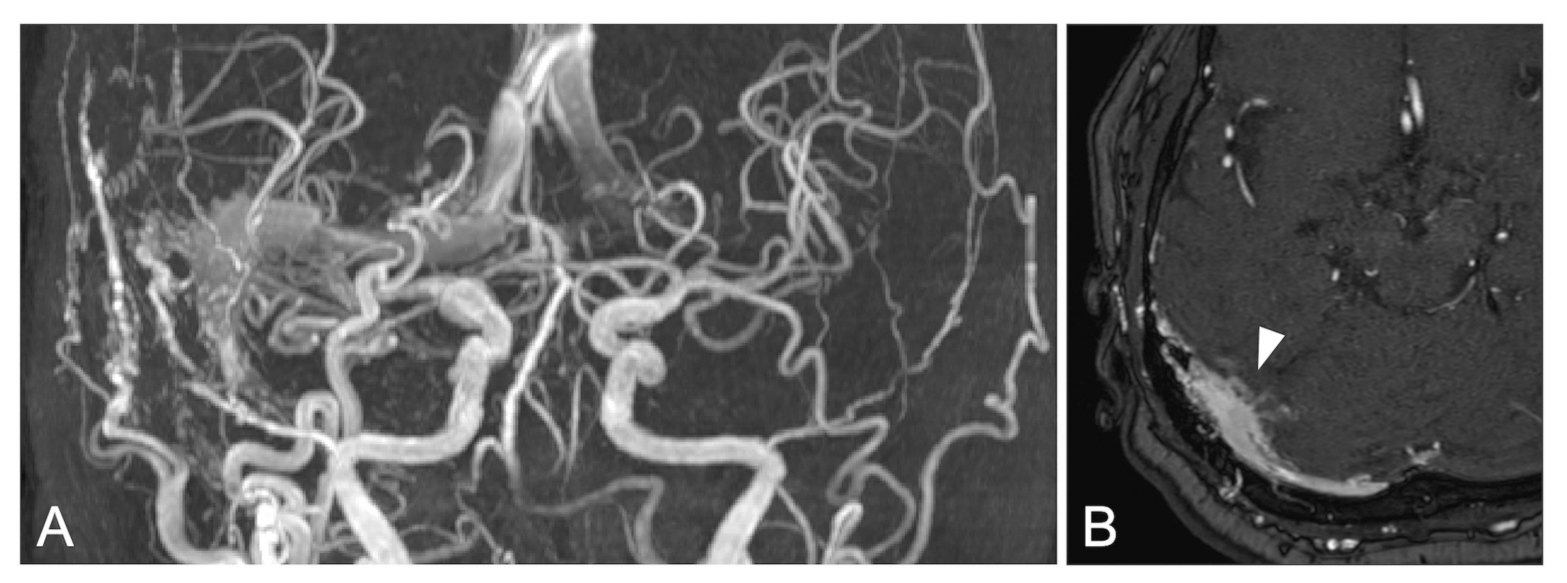
Initial MRI. MRA showing a right transverse-sigmoid sinus dural arteriovenous fistula with both antegrade and retrograde venous drainage (A). The source image of the MR angiography showing cortical venous reflux (arrowhead) (B).

**Figure 2:**
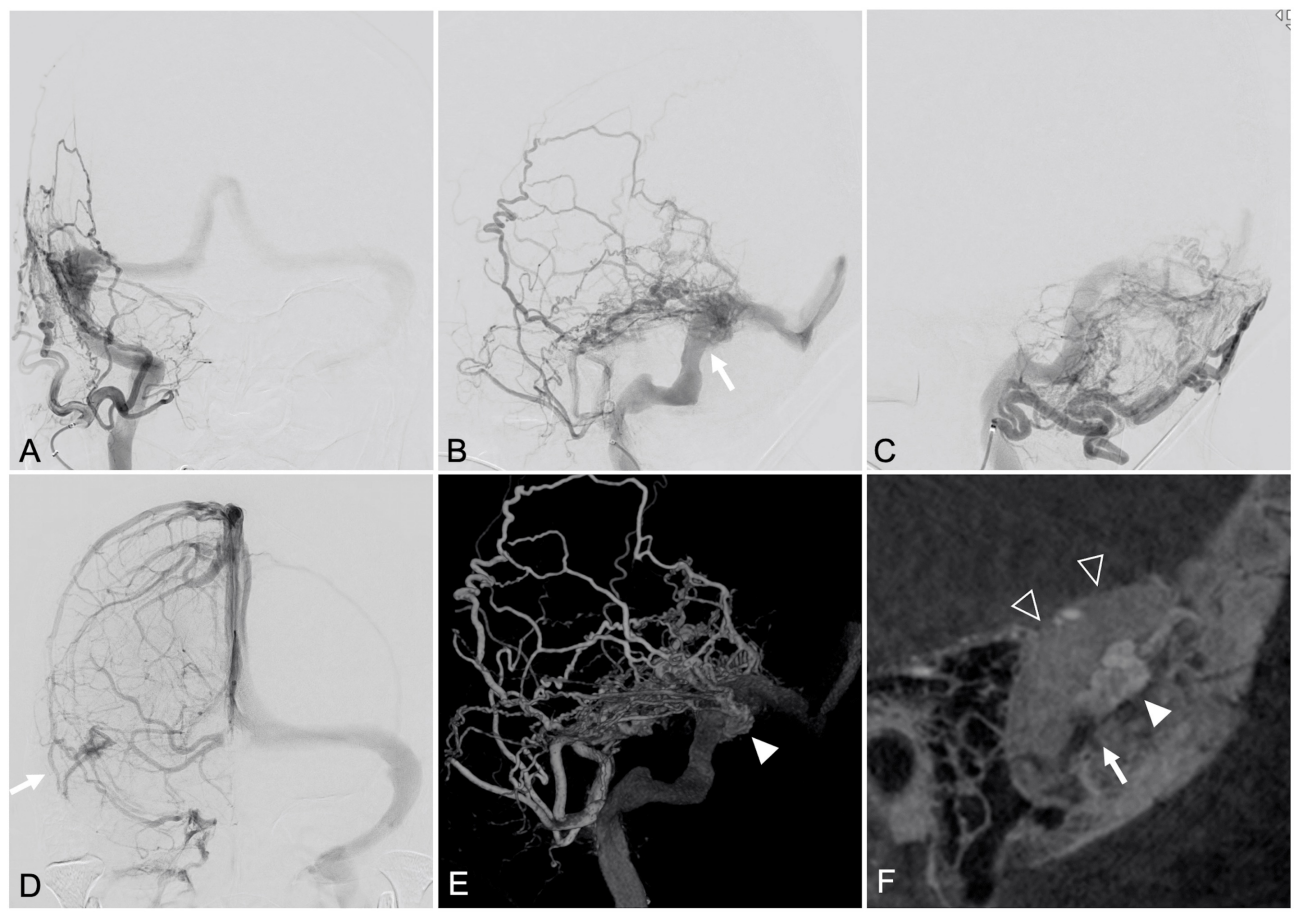
Preoperative angiographic images showing a TSS DAVF with parasinus shunt points. Frontal (A) and lateral (B, C) views of right external carotid artery angiography showing a dural arteriovenous fistula (DAVF) classified as Cognard type IIa/Borden type I, located at the right transverse-sigmoid sinus (TSS). The DAVF is fed by the middle meningeal artery, superficial temporal artery, and occipital artery, with antegrade drainage into the right internal jugular vein and retrograde drainage into the superior sagittal sinus and contralateral transverse sinus. The outlet of the shunted pouch is indicated based on Figure 2F (arrow). (D) The vein of Labbé on the affected side is identified (arrow). (E) Volume-rendered image from rotational angiography showing a shunted pouch (arrowhead) in the parasinus at the posterior-medial aspect of the right TSS. (F) Sagittal reformatted images from rotational angiography showing the shunted pouch (white arrowhead) and its outlet (white arrow) into the main TSS (open arrowhead).

A 76-year-old female patient was admitted to the hospital with a complaint of tinnitus. MRI showed a right TSS DAVF, accompanied by multiple feeders from meningeal and transosseous arteries, as well as antegrade and retrograde sinus flow with tiny cortical venous reflux (Figure 1). Diagnostic angiography revealed feeders from the right middle meningeal artery (MMA), superficial temporal artery (STA), and occipital artery (OA) (Figure 2). A shunt was observed at the junction of the right TSS, with an antegrade drainage to the right internal jugular vein (IJV) and a retrograde drainage to the superior sagittal sinus, contralateral transverse sinus. The cortical venous reflux on MRI was obscured by overlapping feeders and could not be clearly delineated. The vein of Labbé on the affected side drained antegradely into the right TSS. Detailed three-dimensional rotational angiography identified that shunt points were concentrated in the parasinus at the posterior region of the right TSS. The highly tortuous and meandering feeders were solely directed toward and converged on this shunted venous pouch, and its outlet into the main TSS was clearly identified. The maximum diameter of the shunted pouch was approximately 10 mm, and the outlet into the sinus appeared narrow and was located inferiorly. Based on the findings from angiography, this case was classified as Borden type Ⅰ and Cognard type IIa DAVF. Based on the patient’s preference, endovascular treatment was planned.

**Figure 3:**
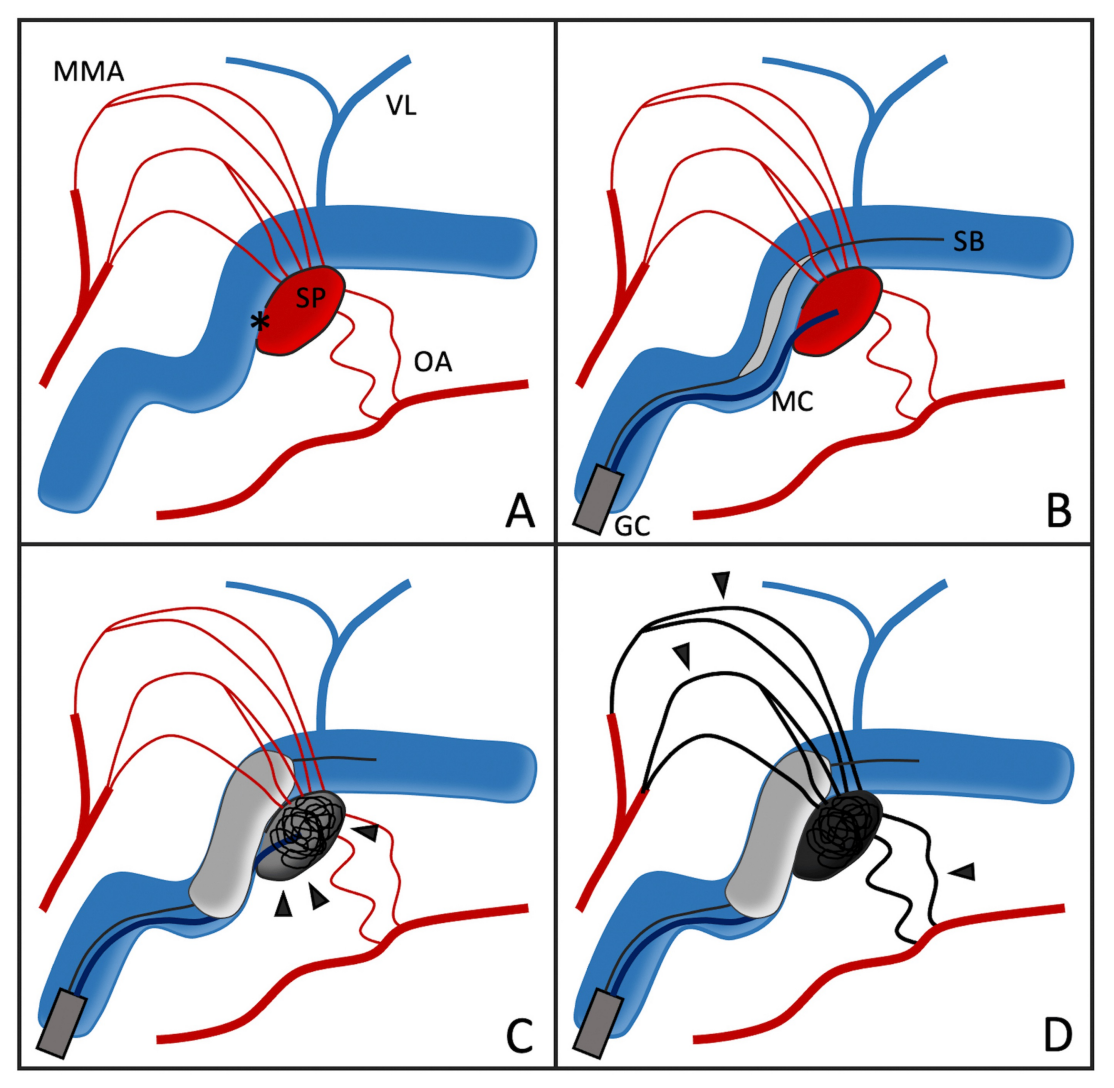
Schematic illustration of targeted transvenous embolization (TVE) using sinus balloon protection. (A) A transverse-sigmoid sinus dural arteriovenous fistula (TSS DAVF) with a distinct shunted pouch (SP). Feeder arteries, such as the middle meningeal artery (MMA) and occipital artery (OA), are directed toward and converged on the SP. The outflow point from SP into the sinus is clearly delineated (*). The vein of Labbé (VL) is illustrated separately to confirm its anatomical separation from the shunt site. (B) A guiding catheter (GC) is placed in the venous system, from which both the microcatheter (MC) and sinus balloon catheter (SB) are advanced. The MC is navigated into the SP, while the SB is positioned across the pouch outflow. (C) With the SB inflated, coils and Onyx are delivered into the pouch (arrowheads) via the MC, achieving localized embolization. (D) Final distribution of Onyx (arrowheads) shows retrograde penetration into multiple feeders and complete occlusion of the shunt. The sinus remains patent, and critical veins such as the VL are preserved.

Sinus occlusion was deemed inappropriate as an initial approach in this case due to the presence of antegrade venous drainage. Balloon-assisted TAE was also considered; however, the presence of multiple, dilated feeders from the MMA, STA, and OA made it difficult to determine the optimal access route, and incomplete embolization was a concern. On the other hand, three-dimensional rotational angiography clearly demonstrated that the outlet of the shunted pouch appeared accessible. Based on these findings, we considered whether microcatheterization of the shunted pouch followed by balloon occlusion of its outlet could enable reverse flow of embolic material from the pouch into the feeders, thereby achieving complete shunt occlusion via transvenous embolization (Figure 3).

**Figure 4:**
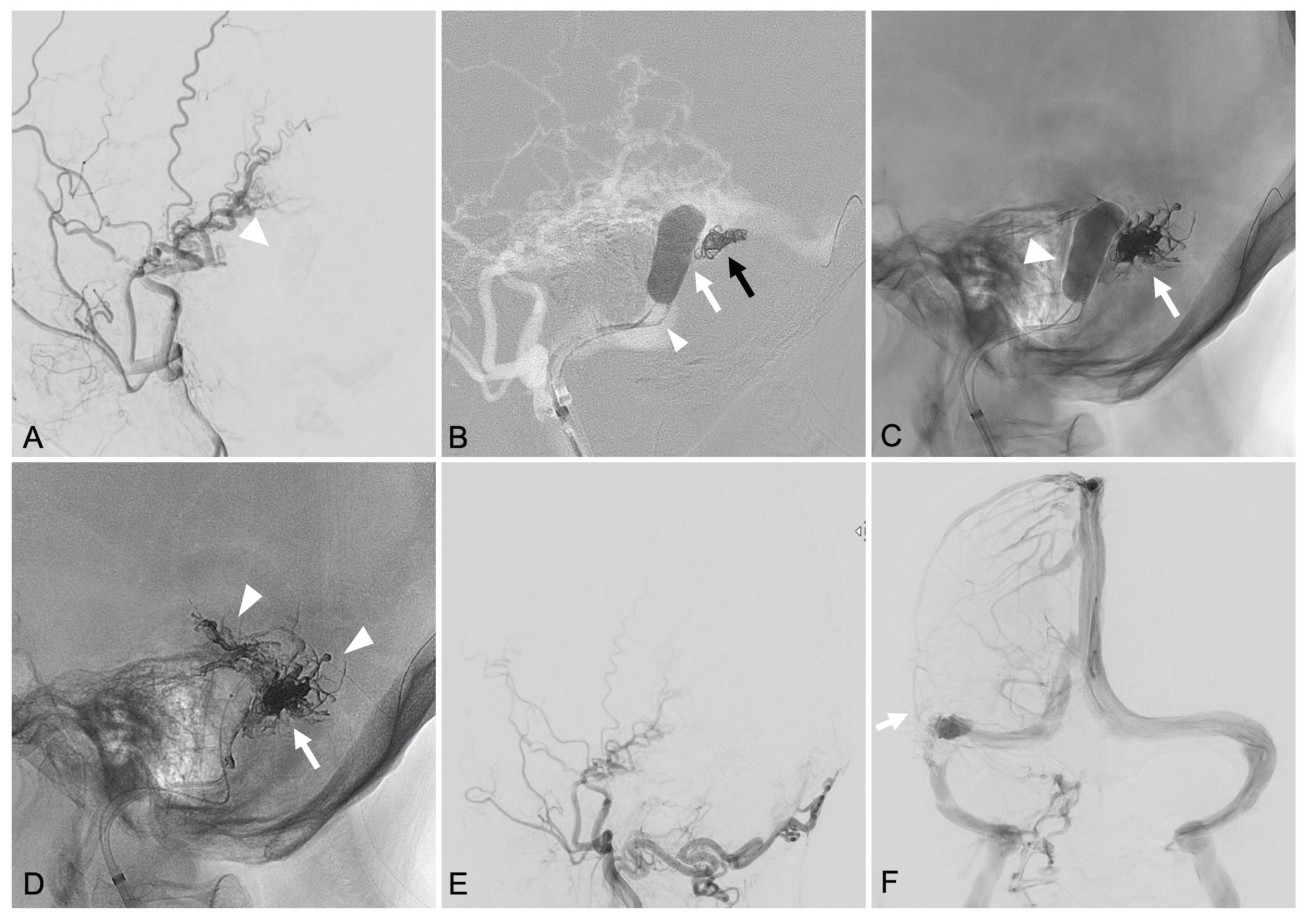
Intra-procedural steps of targeted transvenous embolization using sinus balloon protection using using coils and Onyx. (A) Right external carotid artery angiogram demonstrating disappearance of the arteriovenous shunt under temporary inflation of the SHOURYU2 balloon (arrowhead) at the outlet of the shunted venous pouch. (B) Fluoroscopic image showing a coil scaffold (black arrow) deployed within the shunted pouch following microcatheter navigation (arrowhead) through the outlet of the shunted pouch (white arrow). (C) Fluoroscopic image showing the inflated venous balloon (arrowhead) and the cast of Onyx-18 (arrow) within the shunted pouch. (D) Fluoroscopic image showing the final distribution of Onyx-18 within the shunted pouch (arrow) and retrograde filling into several feeders (arrowhead), confirming complete obliteration of the dural arteriovenous fistula (DAVF). (E) Post-embolization angiogram of the right external carotid artery confirming complete obliteration of the DAVF. (F) Frontal views of the right internal carotid artery angiogram in the venous phase, showing preserved patency of the transverse-sigmoid sinus, as well as normal drainage through the superficial cortical veins and the vein of Labbé (arrow).

Under general anesthesia, an 8F sheath was inserted into the right femoral vein, and an 8F guiding catheter was advanced into the right IJV. Initially, a SHOURYU2 HR 7 × 11 mm balloon catheter (Kaneka Medics, Kanagawa, Japan) was navigated to the right TSS. Under temporary inflation of the SHOURYU2 balloon at the outflow point of the shunted venous pouch, conventional angiography revealed that AVF disappeared (Figure 4). Hence, we decided to perform targeted TVE of the shunted pouch using Onyx (Medtronic, Irvine, CA, USA) under sinus protection. Subsequently, a Marathon microcatheter (Medtronic, Irvine, CA, USA) was navigated into the shunted pouch using a TACTICS PLUS catheter (Technocrat Corporation, Aichi, Japan) as a distal access catheter, and five detachable coils (i-ED Complex SilkySoft; Kaneka Medics, Kanagawa, Japan) were deployed to create a scaffold within the pouch. To prevent Onyx migration into the normal sinus, the SHOURYU2 balloon was inflated to ensure sinus protection while Onyx was injected. The Onyx partially refluxed into some of the feeders from the shunted pouch, leading to complete occlusion of the shunt (Onyx volume: 2.45 mL; injection time: 7 minutes and 30 seconds), while preserving the patency of the sinus and maintaining normal venous drainage through the superficial cortical veins and the vein of Labbé. The postoperative course was favorable, and the patient was discharged without signs of neurologic deficits. No recurrence was observed on angiography performed six months after the procedure.

## Discussion

The treatment of TSS DAVFs presents significant challenges due to the complex angioarchitecture and the potential for serious neurological complications associated with venous hypertension and intracerebral hemorrhage from cortical venous reflux [1,2]. Endovascular treatments, including both TAE and TVE, have been employed with varying success rates [5,6]. Among them, sinus balloon protection has recently been used predominantly in TAE to maintain sinus patency [3,4]. This technique is particularly advantageous in Borden type Ⅰ and Ⅱ DAVFs with a patent sinus, as it facilitates complete DAVF occlusion while preserving the sinus flow. However, the success of TAE depends heavily on the number, diameter, and tortuosity of the feeding arteries. If the embolic material fails to reach the shut points through the feeders, treatment failure can result. Thus, large and complex TSS DAVFs are often difficult to completely occlude under sinus protection. Moreover, TAE carries inherent risks of cerebral infarction or cranial nerve palsy, particularly when the feeders include branches of the MMA, OA, STA, or ascending pharyngeal artery, many of which have anastomoses with major vessels such as the internal carotid and vertebral arteries, and supply cranial nerves [7].

For TVE, sinus occlusion has often been performed for complex TSS DAVFs when TAE is not feasible [6]. However, sinus occlusion may result in elevated venous pressure and subsequent neurological deterioration. Notably, TSS DAVFs often exhibit localized shunt concentrations or *shunted pouches* within the parasinus adjacent to the main sinus [8]. The presence of these shunted pouches allows for a more focused embolization strategy in which only the affected portion of the sinus is treated, preserving overall sinus patency [9-11]. In such cases, coils are commonly used for parasinus occlusion without sinus balloon assistance.

The use of sinus balloon protection in TVE is exceedingly rare, with few reports in the literature. Kerolus et al. introduced the Onyx tunnel technique, in which Onyx is injected under balloon protection to create a controlled embolic tunnel within the sinus, effectively occluding the shunt while sparing the sinus lumen [12]. However, it can be difficult to confirm sinus patency accurately, particularly in cases with long and tortuous anatomy. Moreover, the technique requires precise control of balloon positioning and catheter retrieval timing, particularly when attempting to preserve critical draining veins such as the vein of Labbé. In our case, the shunt points were concentrated within the posterior parasinus region of the TSS. The vein of Labbé was considered to be situated on the opposite side and anatomically separated from the shunt site. The use of sinus balloon protection enabled us to perform a highly targeted embolization, employing both coils and Onyx to effectively occlude the shunt while preserving the patency of TSS and draining veins.

There are several important technical considerations from our case. First, detailed three-dimensional rotational angiography is crucial for identifying the precise location of the shunt points. This enabled strategic catheter placement and optimized the application of sinus balloon protection. Kiyosue et al. also emphasized the importance of three-dimensional rotational angiography in the detailed analysis of DAVF angioarchitecture to guide appropriate treatment strategies [8]. Second, test occlusion by temporary inflation of the balloon catheter of appropriate length at the outflow of the parasinus is important. If the outlet of the shunted pouch cannot be adequately blocked, there is a risk of incomplete embolization and potential migration of Onyx into the main sinus trunk or pulmonary circulation. Indeed, the SHOURYU2 balloon catheter we used can be inflated to a maximum of over 30 mm [13]. Third, the choice of embolic material is a key factor. Onyx, with its non-adhesive and flow-controllable properties, allows for effective filling of the shunted pouch under balloon protection. Pressurizing Onyx within the pouch permits retrograde penetration into feeders, enhancing occlusion and potentially reducing recurrence, similar to the techniques employed in TVE for brain arteriovenous malformations [14]. Although coils have traditionally been used in TVE for shunted pouches, they may require adjunctive TAE due to residual or recurrent shunts [6]. In our experience, Onyx provided a more comprehensive and durable embolization. Additionally, the use of coils as a structural scaffold within the pouch may enhance Onyx stability and effectiveness.

Despite the encouraging results, the technique has limitations. Risks include balloon migration, rupture, or incomplete occlusion if the balloon does not provide sufficient isolation. Moreover, although some residual shunt may remain, additional TAE can be considered as a supplementary approach. Long-term outcomes of sinus-sparing TVE with balloon protection remain under-investigated, and further clinical experience is necessary to validate its safety and efficacy.

## Conclusions

This case demonstrates that targeted TVE under sinus balloon protection may offer a viable alternative to conventional sinus occlusion or TAE with sinus preservation in select cases of TSS DAVFs with parasinus shunt concentrations. Three-dimensional rotational angiography was essential for delineating the detailed structure of the shunted pouch, particularly when considering this TVE approach.
